# Guiding placement of health facilities using multiple malaria criteria and an interactive tool

**DOI:** 10.1186/s12936-021-03991-w

**Published:** 2021-12-03

**Authors:** Kok Ben Toh, Justin Millar, Paul Psychas, Benjamin Abuaku, Collins Ahorlu, Samuel Oppong, Kwadwo Koram, Denis Valle

**Affiliations:** 1 School of Natural Resources and Environment, University of Florida, Gainesville, USA; 2 School of Forest, Fisheries, and Geomatics Sciences, University of Florida, Gainesville, USA; 3 Centers for Disease Control, US President’s Malaria Initiative, Atlanta, USA; 4 Noguchi Memorial Institute for Medical Research, University of Ghana, Legon, Ghana; 5 National Malaria Control Programme, Accra, Ghana

## Abstract

**Background:**

Access to healthcare is important in controlling malaria burden and, as a result, distance or travel time to health facilities is often a significant predictor in modelling malaria prevalence. Adding new health facilities may reduce overall travel time to health facilities and may decrease malaria transmission. To help guide local decision-makers as they scale up community-based accessibility, the influence of the spatial allocation of new health facilities on malaria prevalence is evaluated in Bunkpurugu-Yunyoo district in northern Ghana. A location-allocation analysis is performed to find optimal locations of new health facilities by separately minimizing three district-wide objectives: malaria prevalence, malaria incidence, and average travel time to health facilities.

**Methods:**

Generalized additive models was used to estimate the relationship between malaria prevalence and travel time to the nearest health facility and other geospatial covariates. The model predictions are then used to calculate the optimisation criteria for the location-allocation analysis. This analysis was performed for two scenarios: adding new health facilities to the existing ones, and a hypothetical scenario in which the community-based healthcare facilities would be allocated anew. An interactive web application was created to facilitate efficient presentation of this analysis and allow users to experiment with their choice of health facility location and optimisation criteria.

**Results:**

Using malaria prevalence and travel time as optimisation criteria, two locations that would benefit from new health facilities were identified, regardless of scenarios. Due to the non-linear relationship between malaria incidence and prevalence, the optimal locations chosen based on the incidence criterion tended to be inequitable and was different from those based on the other optimisation criteria.

**Conclusions:**

This study findings underscore the importance of using multiple optimisation criteria in the decision-making process. This analysis and the interactive application can be repurposed for other regions and criteria, bridging the gap between science, models and decisions.

**Supplementary Information:**

The online version contains supplementary material available at 10.1186/s12936-021-03991-w.

## Background

Access to quality health care is an important health system goal [1]. In particular, achieving universal health coverage, which includes access to quality health care, medicines, and vaccines for all, is emphasized in the United Nations Sustainable Development Goals [2]. While there are many factors that contribute to healthcare accessibility, such as cost [3, 4] and social network systems [5], geographic distance or travel time is often recognized as a significant impediment to effective treatment [6–8]. In the case of malaria, accessibility (distance or travel time) to nearby health facilities has long been recognized as a significant factor for controlling malaria burden [9, 10] and a significant predictors of malaria prevalence [11–13]. In this study area in northern Ghana, distance to health facilities, alongside other geospatial predictors such as distance to urban centre, amount of vegetation, and elevation were found to be significantly associated with malaria infection [14, 15]. As a result, adding new health facilities in the district may reduce overall travel time to health facilities and, as a result, may help decrease malaria transmission.

Ghana has been expanding the coverage of the Community-based Health Planning and Services (CHPS) programme through the Ghana Essential Health Intervention Project (GEHIP) [16, 17]. CHPS aims to improve geographical access to health care with an initial focus on remote and rural areas. The primary function of the CHPS program is to train and place community health officers (CHOs), who are nurses with two years of training, in a CHPS zone, which is a demarcated area consisting of a number of under-served communities. The CHOs provide local level health services and health promotion, including reproductive, maternal and childhood services, provision of diagnostic testing and treatment for acute respiratory illness, diarrhoea and malaria, and referral of more severe cases to higher care level [17, 18]. Although CHOs typically travel monthly from nearby health centre or hospital to serve the CHPS zone, a health post known as CHPS compound located at the community is highly desirable. CHPS compounds are equipped with medical supplies and simple diagnostic tests and they serve as a service delivery point as well as the CHO's residence. Ultimately, the CHPS compound enables the community to have prompt access to the CHOs and the services they provide instead of requiring community members to wait for the monthly visit or to travel to a higher health care facility. The programme enjoys broad support from the Ghana Health Service and remains a strong platform for increasing access and availability of malaria case management [19].

While CHPS are not specific to malaria, malaria is strongly associated with many of their evaluation indicators, such as child mortality rates and health of children under five, particularly in highly endemic areas such as northern Ghana [17]. The CHPS programme has been shown to achieve 49% reduction in under-five mortality rates relative to comparison districts [20, 21]. This study focuses on estimating the effects of accessibility to health facilities on malaria, which can then be used to conduct location-allocation analysis, i.e., to determine locations for new health facilities that optimises some criteria [22]. With this analysis, the maximal level of reduction in area-wide malaria prevalence or incidence associated with the placement of hypothetical new health facilities can be determined.

Interactive visualizations can be particularly useful for location-allocation analyses given that these analyses can result in many charts and maps depending on the number of evaluated scenarios and optimisation criteria [23, 24]. Importantly, previous research has demonstrated that interactive information can have greater impact than passive information [25, 26]. Furthermore, the application can also serve as an interactive simulator that allows users to create and explore their own scenarios regarding health facility allocation and how this influences accessibility and health outcomes [27].

In this study, an interactive decision support application is created to enable users to place hypothetical new health facilities (or replace existing ones) and test how the new spatial configuration of health facilities may influence malaria burden. This decision support tool can also be used to determine the optimal location of health facilities. To create the application, the malaria prevalence is modelled using travel time to health facilities among other covariates. Then, the optimal locations for new health facilities are determined based on one of three criteria: overall malaria prevalence, incidence, and the average travel time to nearest health facilities. Based on these results, the optimal vs. the current location of health facilities are compared, revealing two new areas in the district that could substantially benefit from CHPS compounds. The differences arise when using each of these three criteria highligh the importance of using a multi-criteria strategy to optimise the location of health facilities.

## Methods

### Malaria data

**Fig. 1:**
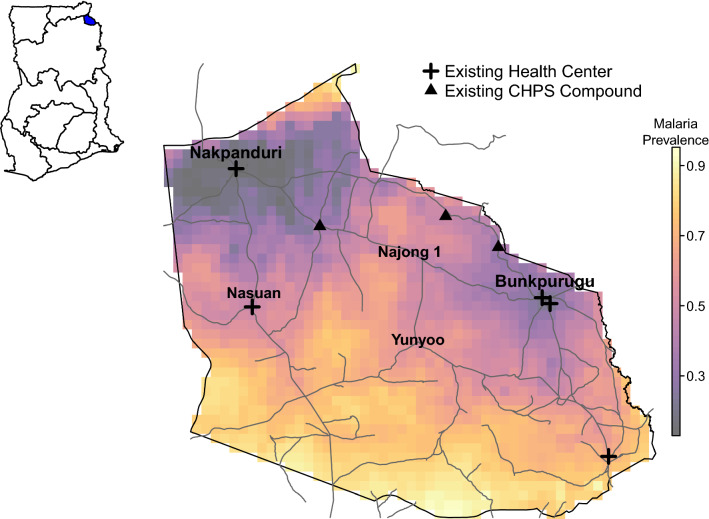
Map of Ghana (top left) and the Bunkpurugu-Yunyoo District (right), which is highlighted in blue on the Ghana map. Roads and existing health facilities are shown together with the predicted malaria prevalence of children under five during the 2012 high transmission season. The prevalence map is from [15]. Nakpanduri and Bunkpurugu are the urban areas in this district, while Nasuan, Yunyoo and Najong 1 are highlighted larger villages

The Bunkpurugu-Yunyoo district (Fig. 1), a rural district in northeastern Ghana bordering Togo, has historically been hyperendemic for malaria and has experienced high transmission during the rainy season. In 2010 and 2013, six cross-sectional household surveys were conducted to assess the impact of an indoor residual spraying (IRS) campaign on malaria parasitemia in Bunkpurugu-Yunyoo. Baseline end-of-rainy season (peak) and baseline end-of-dry season (trough) surveys were conducted in 2010 to 2011. Annual IRS was introduced at the end of the 2011 dry season. Peak and trough season surveys were repeated in 2011–12 and 2012–13, for a total of six surveys [15, 28]. During this period, both IRS and ITN coverage were universal and high throughout the district, indicating little room for improvement using these intervention strategies.

In each survey, households were selected using a multi-stage randomized cluster sampling approach, with clusters sampled with probability proportional to population size and households randomly selected within these clusters. Children under five years of age in these selected households were tested for malaria using both RDT (Rapid Diagnostic Test) and microscopy. Between 1311 to 2040 children were sampled in each survey. The survey datasets were enhanced by the incorporation of remote-sensed data on environmental risk factors for malaria. Details of the survey design and epidemiological evaluation can be found in [15, 28]. Importantly, spatial heterogeneity of malaria prevalence was high, ranging from 19 to 90% across the district [15] (Fig. 1).

In this study, the datasets from the three peak-season surveys in 2010 to 2012 was used, as the overall prevalence, and spatial variation in prevalence, among the three surveys were similar. In these datasets, each sample was geocoded according to their cluster (i.e., children within the same cluster shared the same geographical coordinates). The binary microscopy outcome was used as the malaria infection status (0 = negative, 1 = positive).

### Spatial prediction of malaria prevalence

A Generalized Additive Model (GAM) was used to determine the relationship between malaria prevalence and geospatial covariates, including travel time to the nearest health facility.

### Geospatial covariates

The geospatial covariates used in the model are travel time to the nearest health facility, distance to urban centres, elevation, normalized difference vegetation index (NDVI), and population density (see Additional file 1 for their spatial distribution). These were the most important spatial covariates according to the output of a variable selection procedure adopted in previous studies using the same dataset [14, 15].

The *travel time to the nearest health facility* was calculated using the 2015 global travel time surface based on motorized transport provided by the Malaria Atlas Project [29]. This comprehensive dataset estimates how long it takes humans to travel through a landscape by combining political, infrastructural, and environmental information sources to create a 1 km^2^ resolution “friction surface” for the entire globe. Using this friction surface, the time (in minutes) it takes to travel to the nearest health facility was calculated using the least cost path algorithm [30] with geo-coordinates obtained from Ghana Health Services. This analysis accumulates the cost of moving through each pixel to estimate a more realistic path (and therefore time/distance) than Euclidean distance. The least-cost path analysis was performed using the gdistance package in R [31].

In relation to the other geospatial variables, *distance to the nearest urban centre* (in kilometres) was based on Euclidean distance to the nearest two settlements with population larger than 5000, namely Bunkpurugu and Nakpanduri (Fig. 1). *Elevation* (metres above sea level) was based on the 90 m resolution digital elevation map from Consortium for Spatial Information [32] whereas vegetation was represented using the Normalized Difference Vegetation Index (NDVI) from MODIS, calculated as the maximum monthly index 30 days prior to the survey. Finally, population density was sourced from the five-year stratified WorldPop 2014 population estimates [33].

All covariates, except for distance to health facilities, were raster-based covariates that were interpolated from the nearest four cells of a given coordinate. A summary of all the data used by this study is provided in Table 1.

**Table 1:** Summary of data used for this study

Variable	Description
Malaria Prevalence (Dependent variable)	Calculated based on 2010 to 2012 annual household surveys conducted during peak transmission seasons. Prevalence for each cluster (e.g., village) is the proportion of under five children who tested positive for malaria. Geographical coordinates of each village were available
Location of health facilities	Geographical coordinates of the existing health facilities at the time of the survey were provided by Ghana Health Services
Travel time to nearest health facility	Calculated using the global travel time surface estimated by Malaria Atlas Project. For each location (pixel), the travel time to each health facility was calculated using the least-cost path method and then the shortest travel time was assigned
Distance to nearest urban centre	For each location (pixel), the Euclidean distances to Bunkpurugu and Nakpanduri are calculated; the shortest distance is assigned
Elevation	Extracted from 90 m resolution digital elevation map from Consortium for Spatial Information
Normalized Difference Vegetation Index	Extracted from MODIS
Population density	Extracted from WorldPop

### Model fitting and prediction

The individual malaria infection status was modelled using the GAM with a logit link function. A thin-plate cubic spline was applied only on the elevation, which was found to exhibit a strong non-linear pattern. All other predictors were fitted as linear predictors without splines. This was the best configuration in terms of cross-validation errors based on the preliminary analysis. The survey year was also added to the model as a categorical variable, allowing the intercept to vary from year to year. The GAM was fitted using the mgcv package in R [34].

A 1 km^2^ resolution grid was created over the district and prevalence was estimated for each grid cell. Travel time to health facilities was calculated using the centroid of each grid cell and other covariates were extracted from the rasters using similar methods described in previous section. The fitted GAM model was then used to predict malaria prevalence in each pixel across the district.

### District-wide metrics as optimisation criteria

The location of new health facilities was optimised using one of the three district-wide metrics that are likely to be relevant for decision-makers when placing new health facilities: (a) the expected malaria prevalence of all children under five in the peak season, (b) expected incidence of malaria cases for all ages per person year observed during the high transmission season, or (c) expected travel time to the nearest health facility. The malaria prevalence and incidence metrics are key measures of morbidity, being directly related to strategy objectives listed in Ghana’s Malaria Operational Plan [18].

The predicted prevalence of each pixel was weighted according to its population, yielding the district-wide malaria prevalence:

$$\frac{1}{\sum_{j=1}^{N}{m}_{j}}\sum_{i=1}^{N}\widehat{{p}_{i}}{m}_{i}$$

where $\widehat{{p}_{i}}$ and ${m}_{i}$ are the predicted prevalence and population under five for pixel $i$, respectively, and $N$ is the total number of pixels across in the district.

To calculate the expected incidence per year, the predicted prevalence for children under 5 was first converted to prevalence of children of two to ten years old using the method outlined by [35]. Using the equations from [36], the predicted prevalence of 2 to 10 years old children was then converted to the expected incidence per person-year for children under five, older children (5 to 15 years old) and adults (> 15 years old). Finally, the expected incidence per year over all age groups in the district was estimated using the population of each pixel, and the age structure of the northern region estimated from the 2014 Demographic Health Survey [37].

The travel time to the nearest health facility was estimated for each pixel using the method outlined in ‘Geospatial covariates’ section. Then, the district-wide expected travel time was calculated as the population weighted average of travel time among all pixels. Notice that, differently from malaria prevalence and incidence, the expected travel time to the nearest heath facility is not a malaria specific criterion and, as a result, is not influenced by the GAM results. This optimisation criterion may maximize overall access to healthcare.

### Projecting the impact of new health facilities

One of the main goals of this modelling exercise is to predict changes in malaria prevalence and incidence if new CHPS compounds (onwards referred to as new health facilities) were to be created. The procedure to do this consists of two steps. First, with a given set of coordinates for the proposed new health facilities, the travel time to the nearest health facility is recalculated by rerunning the least-cost path analysis (described in ‘Geospatial covariates’ section) using the new set of health facilities (i.e., the existing and proposed health facilities). Second, the predicted malaria prevalence is updated using the new travel time surface (i.e., the new predicted probabilities represent the projected prevalence if the new health facilities are created). For example, the expected prevalence in a particular location (or pixel) $i$, $E\left({P}_{i}\right)$ would be:

$$E\left(\widehat{{P}_{i}}\right)={\text{logit}}^{-1}\left[\widehat{{\beta }_{0}}+\widehat{{\beta }_{D}}{D}_{i}+\widehat{f}\left({{\varvec{X}}}_{{\varvec{i}}}\right)\right]$$

where ${\beta }_{0}$ is the intercept term, ${D}_{i}$ is the travel time to nearest health facilities and ${\beta }_{D}$ is the corresponding coefficient, $f$ is some spline or linear function and ${{\varvec{X}}}_{{\varvec{i}}}$ represents other covariates. When new health facilities are added, ${D}_{i}$ is expected to either remain the same because the nearest health facility is still one of the existing facilities or decrease because one of the new health facilities is now nearer to the location $i$. The other spatial covariates (${{\varvec{X}}}_{{\varvec{i}}}$) and parameters are assumed to be unchanged. As a result, given that ${\beta }_{D}>0$, expected prevalence will remain the same or decrease when new health facilities are added.

The optimal locations of one up to five new health facilities were determined using this procedure. The optimisation algorithm was ran multiple times, once for each criterion to be minimized and each number of new health facilities. Identifying the optimal location of new health facilities can be a relatively high-dimensional optimisation problem (e.g., five proposed new facilities imply optimisation of ten values [two coordinates per facility]) and can lead to local minima problem. To alleviate this problem, the optimisation was performed using the genetic algorithm in the GA package in R [38].

The optimal locations of health facilities were investigated under two scenarios: adding new facilities to the existing set of health facilities (Scenario 1) and adding new facilities after excluding the existing CHPS compounds (Scenario 2). In the latter scenario, the expected prevalence was computed by updating the travel time to health facilities assuming that the original CHPS compounds did not exist. This scenario can be used to examine whether the current location of CHPS compounds is close to optimal or not.

### Interactive decision support tool

A web-based application was created to let users add hypothetical new health facilities to the district and see how the spatial distribution of malaria risk and the district-wide summary metrics would change correspondingly. The application was developed using shiny package in R [39].

The user is shown a map of Bunkpurugu-Yunyoo District, overlaid by the predicted prevalence, incidence, or travel time map. The user can propose multiple new health facilities by point-and-click interactions with the tool. The prevalence, incidence, and travel time maps, alongside with the predicted district-wide metrics, are updated once all new health facilities are added. Additionally, the user can also visualize the locations of new health facilities proposed by the optimisation algorithm. To do this, the user is required to choose the number of health facilities to be added (from 1 to 5 health facilities) and the optimisation criterion of choice (i.e., minimization of the district-wide malaria prevalence for children under 5 years of age, malaria incidence across all age groups, or travel time). The tool can be accessed at https://kokbent.shinyapps.io/hf-update/.

## Results

### Relationship of malaria prevalence and covariates

**Fig. 2:**
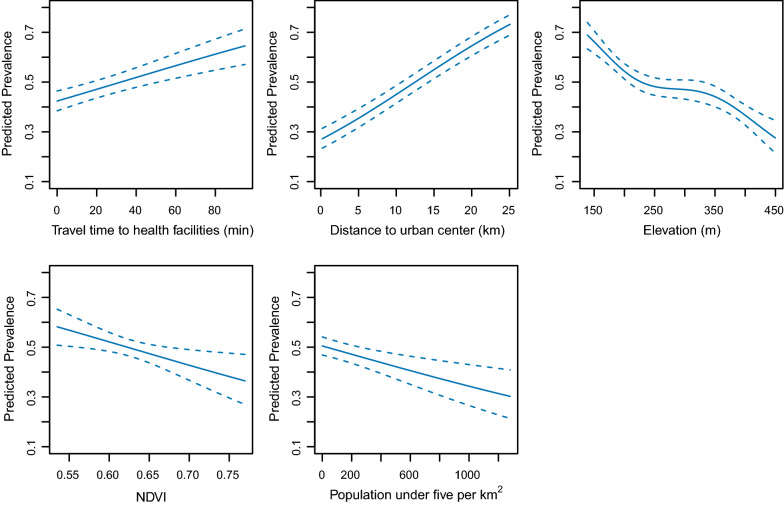
Modelled associations between malaria prevalence and covariates (mean and 95% confidence interval) during peak transmission season

Fitting the GAM model to the data revealed that all of the selected covariates had statistically significant associations with malaria prevalence ($p<0.001$ for all covariates except NDVI with $p<0.05$). Expected prevalence was positively correlated with travel time to health facilities and distance to urban centre, and negatively correlated with the other covariates (Fig. 2). The fitted GAM model explained 65.8% deviance based on McFadden’s R square value calculated under grouped binomial setting.

### Optimal locations for new health facilities

For any optimisation metric, the optimal locations chosen when optimizing for a smaller number of new facilities were a subset of the optimal locations when optimizing for a higher number of new facilities. For instance, let A and B be the optimal locations chosen by the algorithm when optimizing for two new facilities using prevalence criteria. A and B are also optimal locations (or at most 2 km away from the optimal locations found earlier) when optimizing for three to five new facilities using the same criteria. As a result, the locations can be grouped together and the importance of a location group in reducing the selected metric is ranked based on the frequency it appeared in the set of optimal locations when optimised for one to five new facilities.

### Scenario 1: Adding new facilities to the set of existing health facilities

**Fig. 3:**
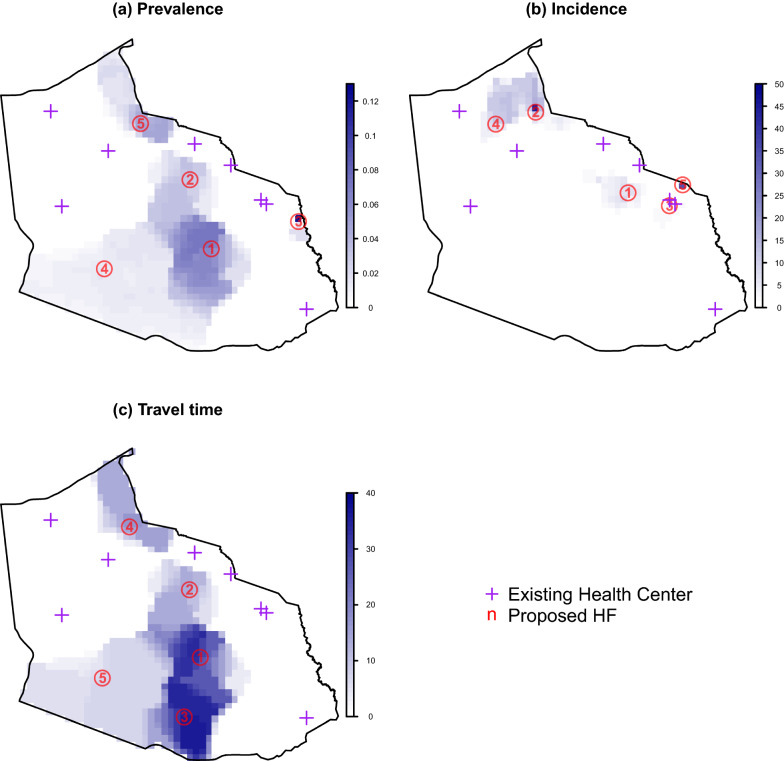
The optimal location of five new health facilities (HFs) that minimizes each of the 3 different district-wide criteria: **a** malaria prevalence of children under five years of age, **b** incidence of all-age malaria cases per 1000 person years observed, or **c** travel time to health facilities (in minutes), under Scenario 1. In this scenario, proposed new facilities are added to the existing facilities. The color of each pixel shows the change in the predicted metrics (without new HFs minus with new HFs). The number associated with each proposed HF indicates their priority: location 1 had highest priority and most reduced the metric used for optimisation, while location 5 was lowest in priority and least reduced the optimisation metric

Optimal locations obtained by minimizing either prevalence or travel time criterion were similar with only one disagreement, and these locations were remarkably different from those based on incidence criterion (Fig. 3). The discrepancy between the optimisation results based on malaria prevalence versus malaria incidence can be attributed to the highly uneven spatial distribution of the population and the fact that the relationship between malaria prevalence and incidence is non-linear. In particular, the addition of a new health facility may decrease prevalence in areas with high malaria prevalence but have little effect on the expected number of cases per year.

When the locations of health facilities (HFs) were optimised using overall prevalence as criteria, adding one up to five health facilities is predicted to reduce districtwide prevalence by 0.3, 0.5, 0.6 and 0.7 and 0.8% (95% CI 0.30 to 0.33 for one HF, 0.74 to 0.81 for five HFs), respectively. Similarly, for district-wide incidence rates during high transmission season, the reductions were equal to 0.35, 0.70, 0.83, 0.95 and 0.99 cases per 1000 person-year (95% CI 0.16 to 0.59 for one HF, 0.58 to 1.40 for five HFs), respectively. Finally, the reductions for travel time per person were 1.5, 2.4, 2.8, 3.2 and 3.5 min. Despite the relatively small changes in districtwide metrics, these proposed health facilities often have strong local impacts. For instance, Fig. 3a reveals that the top priority health facility (i.e., HF 1) reduces prevalence in the surrounding area by 6 to 10%. Importantly, these proposed health facilities do not spatially improve the prevalence and incidence around them in a uniform way (i.e., forming concentric circles around new health facilities), as illustrated in Fig. 3. This is because travel time can differ even when geographical distance is identical, and reduction in travel time to the nearest health facility is dependent on the locations of the other health facilities.

### Scenario 2: Adding new facilities in the absence of the original CHPS compounds

A hypothetical scenario was created in which all current CHPS compounds did not exist and determined the optimal locations for five new health facilities. The results reveal that incidence-based optimisations favoured locations in the northern part of the district, where population density is greatest, while prevalence-based and travel time-based optimisations resulted in one and two locations in the less densely populated central and south regions, respectively.

**Fig. 4:**
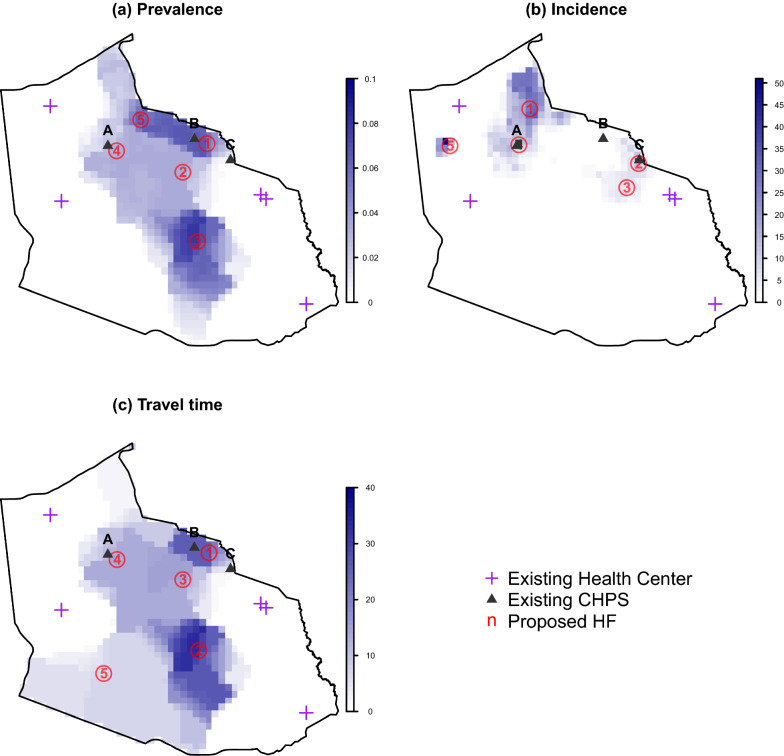
The optimal location of five new health facilities (HFs) that minimizes each of the 3 different district-wide criteria: **a** malaria prevalence of children under 5 years of age, **b** incidence of all-age malaria cases per 1000 person years observed, or **c** travel time to health facilities (in minutes), under Scenario 2. In this scenario, the absence of existing CHPS compounds (labeled as A to C here) was assumed. The color of each pixel shows the changes in the predicted metrics (without new HFs minus with new HFs). The number associated with each proposed HF indicates their priority: location 1 had highest priority and most reduced the metric used for optimisation, while location 5 was lowest in priority and least reduced the optimisation metric

Regardless of criteria, at least two of the optimal locations overlap with the existing CHPS compounds (Fig. 4), suggesting that the position of existing CHPS compounds were generally well chosen to reduce malaria risk. Interestingly, the location around CHPS B (in the map of Fig. 4) ranked the most important using prevalence or travel time criteria, while CHPS C was the second most important based on incidence criteria.

## Discussion

The malaria prevalence during the high transmission season was modelled using travel time to health facilities, distance to urban centres and other environmental factors as predictors. Based on the fitted model, the optimal locations of up to five new health facilities were determined under two scenarios (adding new health facilities to the ones that already existed vs adding new health facilities while assuming that no CHPS compounds existed) and three different optimisation criteria (maximal reduction in district-level prevalence, incidence, or travel time). A web based interactive visualizer and simulator application was created, which effectively incorporates the various components involved in this analysis and helps stakeholders determine the best location for new health facilities.

Past study using the same dataset, Millar et al. [14] found a non-linear relationship between distance to health facility and prevalence. However, it is important to note that these results are not directly comparable to this study because Millar et al. relied on Euclidian distance whereas we relied on travel time. Furthermore, the regression model used by Millar et al. [14] included individual-level covariates (e.g., gender, health insurance, ethnicity, age, occupation, and education), which were not used in this study because incorporating these covariates precludes spatial predictions. Finally, a linear relationship between travel time to nearest health facility and prevalence was used because it yielded the smallest cross-validation errors based on the preliminary analysis.

Since travel time and malaria prevalence are assumed to have a linear relationship on logistic scale, minimizing either parameters yielded very similar results. However, there were some important differences (compare panel A and C in Figs. 3 and 4). These differences likely arise due to the influence of other spatial covariates on prevalence. For instance, in an area predicted to already have low malaria prevalence due to the other spatial covariates, adding a new health facility may substantially decrease local travel time in this area without decreasing district-wide prevalence significantly. Additionally, a new health facility may not improve travel time or prevalence evenly. For example, adding a new health facility in Yunyoo area (Location 1 in Fig. 3c) can reduce travel time much more to the neighbouring area to the west than to the east. The reason for this is that the reduction in travel time and the predicted prevalence is dependent not only on the friction surface, but also on the locations of other health facilities. Patterns like these are not immediately obvious and intuitive and interactive tools are important to help users better understand these relationships.

The optimisation results were substantially different when minimizing incidence, as compared with minimizing population-weighted travel time or prevalence, because of the non-linear relationship between incidence and prevalence. While this relationship was mostly linear for infants and young children, incidence plateaus at moderate and low level of prevalence for older children and adults, respectively [36]. Because of this plateau, reducing prevalence from 80 to 60%, for instance, would not lead to a substantial decrease in overall incidence. On the other hand, in settings with low prevalence, adding new health facilities can both decrease prevalence and incidence [36]. Thus, the spatial optimiser based on the incidence criterion explicitly avoided high transmission areas even if the expected drop in prevalence was large, confining the optimal locations to the northern and eastern regions of the district. In the first scenario, the optimiser even chose the Bunkpurugu town which already had two health facilities as an optimal location. These results underscore the importance of using multiple optimisation criteria to highlight important tradeoffs and assumptions inherent to these criteria. For example, the use of incidence as the optimisation criterion here would be at odds with current healthcare policies that aim to reduce barrier to geographical access to health care.

Despite these differences, there were some general agreements in optimal locations of health facilities when using these objectives. Under scenario 2, two out of three existing CHPS compounds matched the optimal locations found by the algorithms. These results highlight that the current positions of CHPS compounds were nearly optimal based on the model. Nevertheless, the model often places a single health facility in proximity of the two existing CHPS compounds near the eastern border (CHPS B and C in Fig. 4). This study focused purely on accessibility but not availability, which is another important access dimension of healthcare system [40]. CHPS zones are drawn according to population and a CHPS compound may not be available to villagers from other CHPS zones even if they can access it. In addition, the capacity of the health facilities was not considered in this exercise. Two CHPS compounds with small capacity that are close to each other may be necessary if the communities they serve are highly populated. Finally, areas close to Yunyoo and Najong 1 (see Fig. 1) would strongly benefit from new health facilities (Location 1 and 2 in Fig. 3a). Both locations experienced moderate-to-high malaria prevalence during rainy season and had relatively high travel time to nearest health facilities given their population density. In the context of malaria control, children health and survival, and the importance of reducing barriers to healthcare, this analysis suggests that these locations could be prioritized for new CHPS compounds.

Intervention based on reducing travel time by adding new health facilities is likely insufficient: while they can reduce malaria risk in their vicinity, their impact on district-wide metrics is minimal. Even with the most optimal placement of health facilities, five additional health facilities are expected to reduce district-wide prevalence and incidence by less than 1% and less than 1 case per 1000 person-year observed (See Sect. 3.2.1). It is important to note that creating new health facilities is not the only way to reduce travel time and, as a result, decrease malaria risk. Improving travel time by having better infrastructure and better outreach of the community-based health care system may also play important role in ensuring that people have better access to healthcare.

This analysis is done based on the best estimates based on the data available with some important limitations and assumptions. For example, the proof-of-concept decision support tool is based on malaria data from 2010 to 2012, and more up-to-date information is needed to confirm if its recommendations are still valid. Importantly, this study focuses on statistically learning from the interventions that are captured by the high-quality datasets from 2010 to 2012. As a result, evaluation and comparison with other potential interventions are not conducted as they would require a different modelling approach (i.e., simulation-based models).

Additionally, correlation does not imply causation: having a new health facility in a particular location does not necessarily and automatically reduce the malaria prevalence in its vicinity. The travel time to health facility may serve as a proxy of built environment that is not captured in the other covariates (e.g., distance to urban centre, vegetation index and population density). However, the relationship between distance to health facility and malaria is strongly supported by current literature. For example, early diagnosis and treatment of malaria is well known to contribute to reduced disease transmission and malaria death [41, 42], and accessibility to healthcare, which includes proximity to health facility, is widely acknowledged to decrease malaria prevalence [43–46]. Moreover, the travel time to health facility remains a strong predictor of malaria prevalence in the Bunkpurugu-Yunyoo district even after accounting for wide suite of other individual and geospatial covariates [14, 15]. Taken together with the finding that distance to CHPS and health centres was stronger predictor than that of distance to health centres alone, location of health facilities does influence malaria transmission in a way that is independent of built environment.

It is assumed that the associations between malaria prevalence and the other spatial covariates (i.e., distance to nearest urban centre, elevation, vegetation index, and population density) will remain unchanged, which may not be the case. Moreover, since malaria incidence in the Bunkpurugu-Yunyoo district was not readily available, the observed prevalence was converted to incidence based on a model ensemble analysis which relied on data from 30 sites in Sub-Saharan Africa collected from 1981 to 2011, only one of which was from Ghana [36]. These sites may not be necessarily representative of the study district and using the formula to convert prevalence to incidence may not have adequately captured the relationship between these malaria indicators for this study site. Moreover, uncertainties in this study were calculated based on fitting the GAM: uncertainty with respect to model choices (e.g., linear vs non-linear effects) and uncertainty regarding the conversion of prevalence to incidence were not accounted. Another important limitation is the lack of information about the location of health facilities on neighbouring districts. Consequently, it is implicitly assumed that there are no health facilities close to the districts borders or that people do not go to them, particularly when that entails crossing the eastern international border to Togo.

The global surface friction used here was created based on the fastest mode of transportation for each location [29], without accounting for seasonal variation. As a result, the resulting travel time should not be interpreted as the actual travel time a specific person would take to travel to the nearest health facility but rather just a better measure of distance when compared to Euclidean distance. Although a 2020 travel time surface with and without motorized transportation was recently published [47], model fit was worse using these updated travel time surfaces. The 2015 global surface friction better matches the accessibility patterns in the 2010–2013 malaria datasets. For this reason, all results reported here are based on the 2015 global travel time surface.

The optimal locations determined here may seem attractive on the paper but may be impractical or unsuitable on the ground. For this reason, it is important for decision-makers and other stakeholders to be able to interact with the model themselves (e.g., via an interactive decision support tool) to explore other locations that may be more feasible and yet close to optimal. Furthermore, the analysis framework in this study can be extended to other malaria or non-malaria criteria if the corresponding datasets are available. For example, malaria deaths, which is an important Global Technical Strategy goal for malaria [48], can be incorporated to the framework if these data are available at the household or village level. The benefit of the tool created in this study is likely to be more limited in settings where malaria is not the top priority. However, it is important to emphasize that this tool can be extended to other target indicators of the CHPS programme such as diarrhoea and acute respiratory illness [49] if the corresponding data (e.g., prevalence data with spatial coordinates and location of health facilities) are available. Furthermore, even if data on these other diseases are not available, the malaria-focused tool can still be highly useful because it can be used together with other maps and expert opinion on these other diseases to determine the optimal location of health facilities.

## Conclusions

Instead of only using statistical models to estimate the well-documented relationship between travel time to health facilities and malaria transmission, scenario analysis within the interactive tool is a much more informative way to communicate the implications of the modelling results. In this case study, location-allocation analysis can leverage a relatively standard risk factor model to create actionable decisions by integrating other pieces of information such as population distribution, estimated travel time, and prevalence to incidence conversion. Importantly, this analysis, together with the use of multiple optimisation criteria, can uncover patterns that are not immediately obvious when gleaning any single piece of information. For example, prioritizing incidence over prevalence might have overlooked the barrier to healthcare access for communities in the southern, less populated, and higher burdened areas of Bunkpurugu-Yunyoo District. Additionally, although travel time to health facilities is an important spatial predictor to malaria risk, this analysis indicates that reducing average travel time by having more health facilities per se may only have little effect to the district-wide malaria conditions.

The complexity of such analysis can be efficiently communicated through a web based interactive decision support tool. Instead of static maps and figures, an interactive tool allows stakeholders to experiment with their own choices of locations with a few mouse clicks. This is particularly important because modeler-initiated analysis like this can rarely account for all the criteria and constraints that a decision maker may consider. As a result, the interactive tool enables decision makers to use inference from the statistical model and optimisation results in conjunction with any additional criteria that they might have to make an informed decision, helping to bridge the gap between decision makers and modelers. Adding new optimisation criteria (e.g., focusing on other endemic diseases or health conditions) would be relatively straightforward once the statistical framework and interactive tool are created. The code for the interactive tool is publicly available (at https://github.com/kokbent/byd-hf-update) because the tool can be repurposed for other regions and criteria with appropriate data, and potentially be enhanced by incorporating cost information (as in [50]).

Creation of an interactive tool to support decision making process requires stakeholder participation and this proof-of-concept tool is by no means a finalized product. However, by allowing others to visualize a statistical or mathematical model in a straightforward fashion, a prototype like this can facilitate discussions, consensus building and lead to future iteration of a tool that is more impactful. Importantly, public health scientists can now, more than ever before, create interactive applications without deep knowledge in computer science. Harnessing such technologies will be important in bridging the gap between science, models, and decisions.

## Supplementary Information

**Additional file 1.** Spatial distribution of the geospatial covariates and prediction uncertainty of prevalence. Figure of the spatial distribution of the five covariates chosen to model the malaria prevalence of Bunkpurugu-Yunyoo district. Additional figure of prediction uncertainty of malaria prevalence based on the GAM model.

## Data Availability

The scripts used in this analysis and for the creation of the interactive tool are available at https://github.com/kokbent/byd-hf-update. The Shiny interactive application is available at https://kokbent.shinyapps.io/hf-update/. The dataset used in this study is available from the authors upon request.

## Abbreviations

CHPS: Community-based Health Planning and Services
GAM: Generalized Additive Model
GEHIP: Ghana Essential Health Intervention Project
HF: Health Facility
IRS: Indoor Residual Spraying
NDVI: Normalized Difference Vegetation Index
RDT: Rapid Diagnostic Test
