# Supplementary material for: Guiding placement of health facilities using multiple malaria criteria and an interactive tool
Source: Malar J. 2021 Dec 3;20:455. doi: 10.1186/s12936-021-03991-w (PMC8641186; doi:10.1186/s12936-021-03991-w)

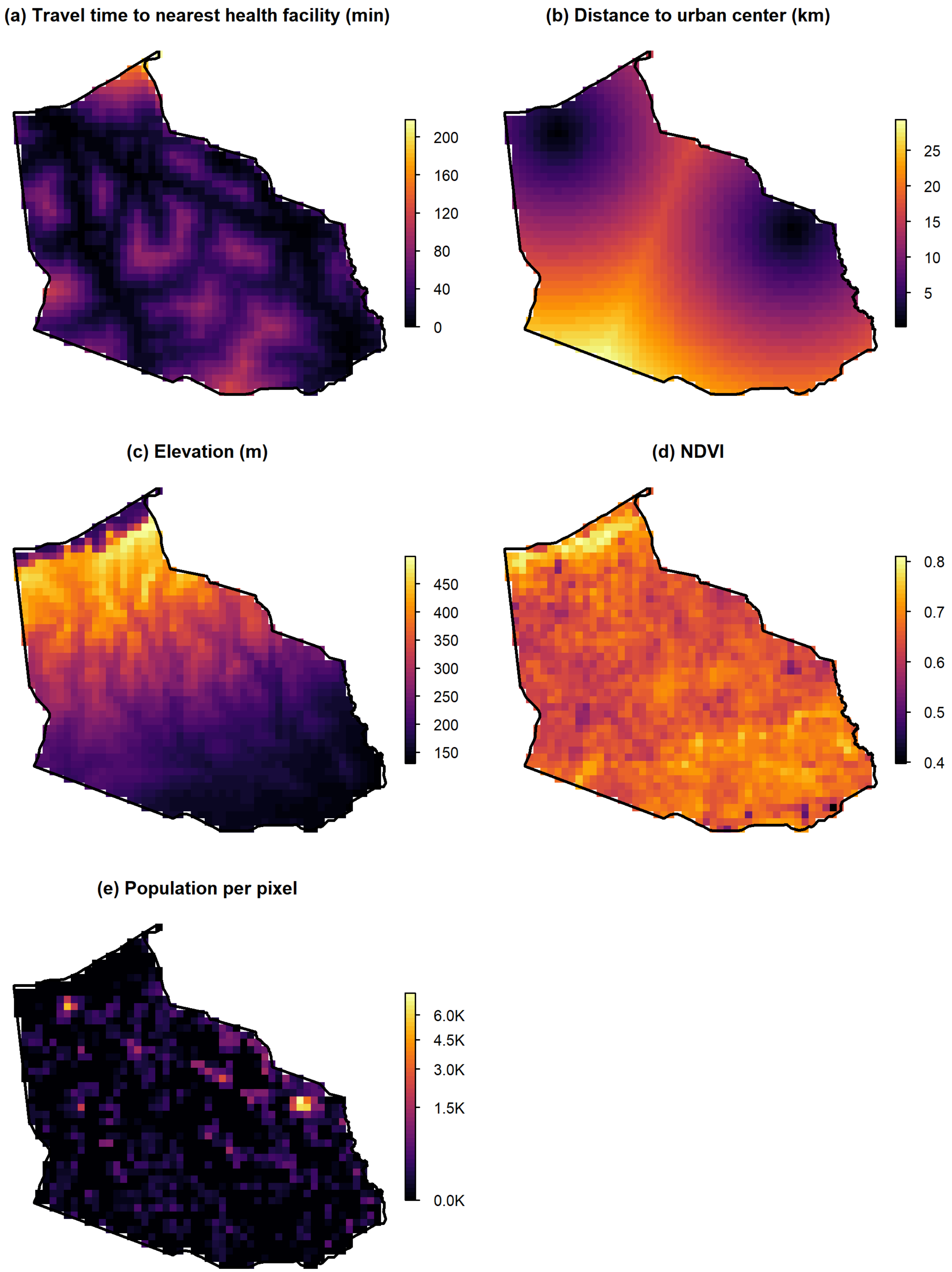
(a) Travel time to nearest health facility (min)
200
160
120
80
40
0
(b) Distance to urban center (km)
25
20
15
10
5
(c) Elevation (m)
450
400
350
300
250
200
150
(d) NDVI
0.8
0.7
0.6
0.5
0.4
(e) Population per pixel
6.0K
4.5K
3.0K
1.5K
0.0K

Width of confidence interval of predicted malaria prevalence

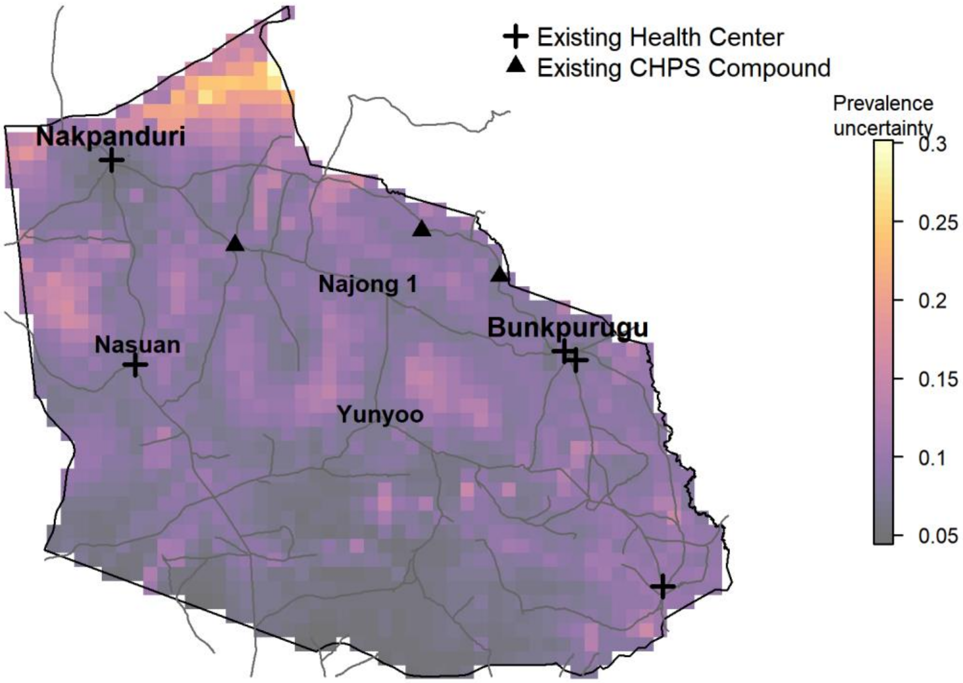

Supplement: Supplementary file 1 — Additional file 1. Spatial distribution of the geospatial covariates and prediction uncertainty of prevalence. Figure of the spatial distribution of the five covariates chosen to model the malaria prevalence of Bunkpurugu-Yunyoo district. Additional figure of prediction uncertainty of malaria prevalence based on the GAM model. [file 12936_2021_3991_MOESM1_ESM.pdf]
